# Carcinosarcoma of the Thyroid Gland

**DOI:** 10.1155/2015/494383

**Published:** 2015-02-03

**Authors:** Mehmet Fatih Ekici, Cengiz Kocak, Zülfü Bayhan, Sezgin Zeren, Faik Yaylak, Mehmet Hüseyin Metineren, Fatma Emel Kocak

**Affiliations:** ^1^Department of General Surgery, Evliya Celebi Training and Research Hospital, 43000 Kütahya, Turkey; ^2^Department of Pathology, Faculty of Medicine, Dumlupinar University, 43100 Kütahya, Turkey; ^3^Department of General Surgery, Faculty of Medicine, Dumlupinar University, 43100 Kütahya, Turkey; ^4^Department of Biochemistry, Faculty of Medicine, Dumlupinar University, 43100 Kütahya, Turkey

## Abstract

*Introduction*. Surgeon has significant role in the management of such rare and controversial clinical entities related to thyroid gland. In this case report we have presented an elderly patient with rapid enlargement in thyroid that was related to carcinosarcoma. *Case Presentation*. A 60-year-old lady was presented with rapid enlargement of the thyroid gland. A fine needle aspiration of the nodule in right lobe was performed several weeks before presentation to our clinic. End diagnosis was a papillary carcinoma of the thyroid with spindle cell component. Thus the nodule was recognized to be a carcinosarcoma. *Conclusion*. Thyroid surgery merits a multidisciplinary approach. Indeed the surgeon should make a conclusive decision in some controversial and rare clinical entities such as carcinosarcoma.

## 1. Introduction

Thyroid fine needle aspirations and histopathological examinations after thyroidectomy are the mainstay in thyroid surgery. However, a detailed history and physical examination are the primary of patient evaluation and management. Previously Hatipoglu et al. have reported a case of anaplastic carcinoma in a geriatric patient who was presented with pain in mid mandibular region. In this case report, the authors have indicated the importance of thorough and systemic medical history taking [1]. Any abnormalities in the thyroid such as recent enlargement or pain should direct the surgeon to further investigation. Functional assessment and morphology of the thyroid gland should be documented. A basic knowledge of the appropriate laboratory tests, imaging procedures, and procedures for tissue sampling should not be ignored. Fine needle aspiration and cytology are recommended to select the patient for thyroidectomy [2]. However, it is not free of complications such as hematoma or pain. Histopathological examination differentiates benign or malign conditions. Rarely unusual findings such as secondary carcinomas or rare types of primary thyroid cancer may be reported. Thus, a multidisciplinary approach is essential in the management of thyroidectomy patients.

In this case report, we have presented an elderly patient with rapid enlargement in thyroid that was related to carcinosarcoma. The aim was to discuss the role of the surgeon in the management of such rare and controversial clinical entities related to thyroid gland.

## 2. Presentation of Case

The patient was a 60-year-old lady and was presented to our clinic with a rapid swelling in neck, dysphagia, and sore throat several days after thyroid fine needle aspiration procedure. She has declined dysphagia, dyspnea, or any voice alterations prior to this aspiration. This cytology was reported to be benign. On physical examination, a thyroid nodule (noted as 5 cm) was palpated. Bilateral cervical lymph nodes were not palpable. TSH was (2.5 uU/mL) in normal ranges (0.3–4.0 uU/mL). Anti-TPO and anti-TG were 600 IU/L and 27 IU/L, respectively. Thyroid ultrasonography has demonstrated heterogeneous nodules in the right thyroid lobe with rough calcifications. Right lobe was 43 × 44 × 68 cm and the left was 16 × 17 × 42 mm in size. Dominant nodule was on the right lobe with a 43 × 30 mm size and a cystic view was reported due to hemorrhage. Another nodule (noted as 20 × 17 mm) was reported inferior to this one. Scintigraphy mCi99mTc-pertechnetate has demonstrated a hypoactive nodule in the right superior-middle section of the thyroid gland and a minimal increase uptake in the inferior section. The patient was treated with total thyroidectomy. Perioperative and postoperative course were uneventful. Carcinosarcoma or papillary carcinoma with spindle cell component was reported after histopathological examination.

**Figure 1:**
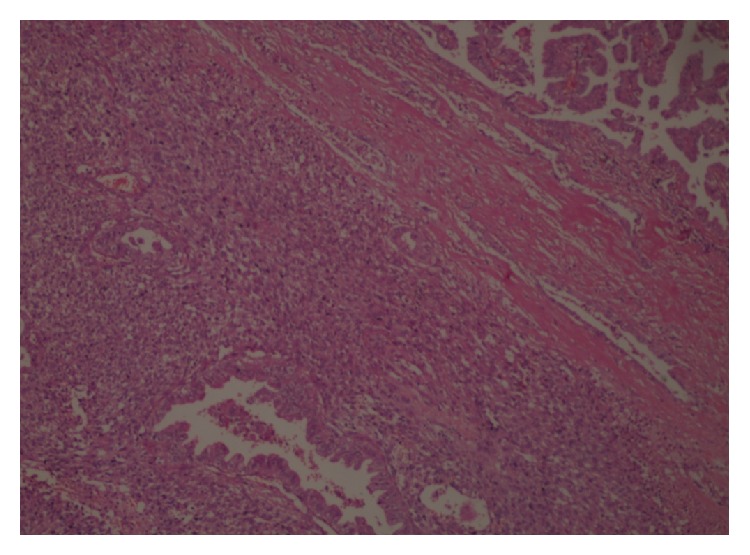
Bimorphic tumour with papillary carcinoma and sarcoma components, black and blue arrows, respectively (H&E ×100).

**Figure 2:**
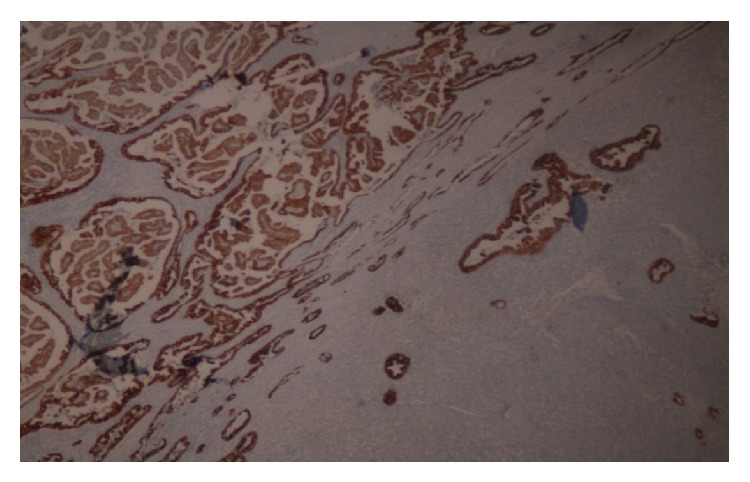
Positive immunoreactivity with HBME-1 antibody in the papillary carcinoma component (×40).

**Figure 3:**
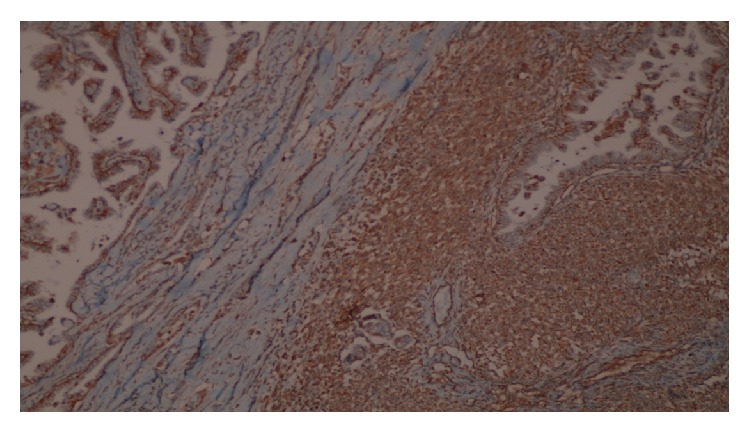
Positive immunoreactivity with vimentin antibody in the sarcoma component (×40).

Surgical margins were clear and intrathyroidal tumor was without capsule invasion. Histopathology has documented that E-cadherin, HBME-1, galectin-3, pancytokeratin, and keratin-19 staining were positive in papillary component (Figures 1 and 2). In addition, spindle cell components were recognized with vimentin positivity (Figure 3).

Patient was scheduled for debulking surgery one month after primary surgery. Chemotherapy and radiotherapy were not scheduled. The overall survival was only two months.

## 3. Discussion

In this case, we observed that compliance to basic knowledge in the management of the patients with any enlargement or nodules in thyroid gland is critical for the patients favor and from various points for surgeons. As it is known fine needle aspiration is not free of complications such as hematoma and it may contribute in the surveillance of the nodules in the thyroid gland [3]. The cytology was reported to be benign but rapid enlargement of the nodule with rough calcifications was the primary indication for surgery. Total thyroidectomy was performed due to additional suspicious surgical findings such as a hard lobe with dense extensions to surrounding tissues. The intention was to prevent a redo surgery for the left lobe. Absence to demonstrate cervical lymph node involvement in preoperative workup was the reason for not performing cervical lymph node dissection. However, our surgical margin was narrow and focal lymphovascular invasion was reported. The conclusive diagnosis of the nodule was available with a thorough and systemic histopathological examination with immunohistochemistry.

Papillary carcinoma of the thyroid gland is the most common endocrine tumor with well-known clinical and histopathological findings. Early lymphatic involvement is not rare and rare variants have been reported previously [4]. Anaplastic carcinoma of the thyroid gland is a rare form according to nomenclature of World Health Organization [5]. Previously it was advocated that it may be developed by degeneration of a well-differentiated thyroid carcinoma. Recently, Agrawal et al. have advocated that, with the presence of both malignant epithelial and mesenchymal cells, carcinosarcoma should be considered a distinct entity from anaplastic carcinoma as WHO clubs have presented. Unlike previous reported cases, they have reported a combination of papillary carcinoma and undifferentiated sarcoma of the thyroid carcinoma which has never been reported in the literature [6]. Here we reported a case with papillary carcinoma with spindle cell component of the thyroid gland. Our observation was comparable with this recent observation. However, we did not observe cervical lymph nodes. Our case may implicate that the recent avocation may be true. However, an intermediate period of dedifferentiation of the primary well-differentiated tumor with epithelial origin may not be excluded. The absence of the cervical lymph nodes in our case was not confirmed with histopathological examination and postoperative imaging as the patient was dropped from follow-up.

## 4. Conclusion

It is clear that best clinical and surgical approach should include steps indicated in various thyroid guidelines. This practice is important in the management of the patients with any thyroid pathology. Routine histopathological examination may help to diagnose. Some controversial clinical findings such as carcinosarcoma or papillary carcinoma with spindle cell component may be clarified with multidisciplinary approach and immunohistochemistry.
